# Antihyperglycemic Activity of Various Fractions of *Cassia auriculata* Linn. in Alloxan Diabetic Rats

**DOI:** 10.4103/0250-474X.41461

**Published:** 2008

**Authors:** S. J. Surana, S. B. Gokhale, R. B. Jadhav, R. L. Sawant, Jyoti B. Wadekar

**Affiliations:** P. D. V. V. P. F's College of Pharmacy, Vilad Ghat, Post MIDC, Ahmednagar-414 111, India

**Keywords:** *Cassia auriculata*, antidiabetic activity, alloxan-induced diabetes, hydromethanolic extract

## Abstract

Present work describes the potent antidiabetic fraction from flowers of *Cassia auriculata* Linn. Hydromethanolic extract along with its ethyl acetate and n-butanol fractions were evaluated for antidiabetic activity in alloxan-induced diabetes in rats. The n-butanol fraction exhibited significant reduction (p<0.001) in blood glucose levels and was also found effective in restoring the blood lipids and proteins to normal level. The activity was found comparable with standard drug phenformin. The hydromethanolic extract and its fractions were subjected to preliminary qualitative chemical investigations which indicated the presence of phenolic compounds, carbohydrates, tannins, steroids and amino acids.

Diabetes Mellitus is a fast growing medical problem in affluent societies and critically attack on metabolic activity of patient. As per the WHO, Diabetes Mellitus is a heterogeneous metabolic disorder characterized by common feature of chronic hyperglycaemia with disturbance in carbohydrate, fat and protein metabolism 1. In India, this disorder is on alarming condition as compared to most of the developed countries. Despite advances in understanding of the disorder and the management, the mortality and morbidity due to this disease is increasing 2. The focus has been shifted to treat the various ailments through plant-derived drugs due to their safety, efficacy, cultural acceptability and lesser side effects.

*Cassia auriculata* Linn., a member of genus Cassia belonging to family *Caesalpiniaceae* is commonly known as Tanner's Cassia 3. It is a shrub found throughout southern, western and central India 4. The various parts of the plant has been reported to posses a number of therapeutic activities to manage disease states like leprosy, asthma, gout, rheumatism 5 and diabetes 6. It is also used as antipyretic, antiulcer and in the treatment of skin infections 7. The flower has been reported to contain flavonoids, proanthocyanidins and β-sitosterol 8–9.

In folk remedies, flowers of *Cassia auriculata* Linn. are proposed to have antidiabetic activity. From literature survey, it was evident that the aqueous extract of flowers has been reported for its antidiabetic activity in streptozotocin-diabetic rats at a dose of 0.45 g/kg body weight 10. In the present investigation an attempt has been made to find out the potent fraction responsible for the said activity at a dose of 0.20 g/kg body weight. Thus, the present work describes effect of the hydromethanolic extract and its fractions on the blood glucose, total protein, cholesterol and triglyceride level in alloxan-diabetic rats.

The flowers of *Cassia auriculata* Linn. were collected from the premises of Shirpur situated in Dhule district of Maharashtra during March 2005. The crude drug was authenticated from the Botany Department, L. K., Dr. P. R. Ghogrey Science College, Dhule (MS). Herbarium specimen was deposited in Botany Department for future reference. The flowers were dried under shade and crushed into coarse powder. Coarse powder was extracted by cold maceration method using methanol:water (1:1). The extract thus obtained was concentrated in rotary flash vacuum evaporator and further dried in vacuum oven. The hydromethanolic extract was subjected to fractionation with ethyl acetate and n-butanol. The fractions were dried in vacuum oven and utilized for animal studies.

In house bred Wistar male rats weighing between 180-220 g were utilized for study. Animals were housed in polypropylene cages and given standard pellet diet and water *ad* libitum. The ethical clearance was obtained from the Institutional Animal Ethical Committee (Approval no. 152/02/C/CPCSEA). Diabetes was induced in rats by intraperitoneal injection of alloxan monohydrate 11 (S. D. Fine Chemicals, Mumbai) in ice-cold citrate buffer, pH 4.5 at a dose of 0.12 g/kg. The diabetic state was confirmed 48 h after alloxan injection by weight loss and hyperglycemia 12. Rats with blood sugar levels 200-350 mg/dl were selected for the study.

The rats were divided into six groups each carrying six animals. Group I served as diabetic control and received 0.3% w/v CMC, orally and alloxan. Group II served as positive control and received standard drug phenformin (0.04g/kg). Group III received hydromethanolic extract of flower (0.20g/kg). Group IV received n-butanol fraction of hydromethanolic extract (0.20g/kg). Group V received ethyl acetate fraction of hydromethanolic extract (0.20g/kg). Group VI served as normal untreated group. The treatment was continued for 8 d by administering the respective fractions/drug in 0.3% w/v CMC. On ninth day of the treatment, blood samples were collected by puncturing the retro-orbital plexus under mild ether anesthesia and kept aside for ½ h for clotting. Serum was separated by centrifuging the samples at 6000 rpm for 20 mins and stored in the refrigerator until analyzed. The serum was analyzed for blood glucose level (GOD-PAP method) 13, total protein (Biuret method) 14, cholesterol (CHOD-PAP method) 15 and triglycerides (enzymatic method) 16 according to the kit manual. The quantitative measurements were made on six animals in each group and the values of biochemical estimations are expressed as mean±SE. The data obtained were subjected to one-way ANOVA followed by Bonferroni multiple comparison test 17.

Alloxan-induced β-cell cytotoxicity is well established animal model for investigating antidiabetic activity. From the results, it is evident that hydromethanolic, n-butanol and ethyl acetate fraction shown significant (*P* < 0.001) reduction in blood glucose level. However n-butanol fraction was highly effective and results are comparable with that of reference drug, phenformin (Table 1). Alloxan treated rats shown substantial weight loss and also affect carbohydrate, lipid and protein metabolism. All three extracts were found to be effective in restoring the body weight of animals to the normal value. Elevated blood lipids especially cholesterol and triglycerides as well as reduction of protein level are other indicators of diabetic condition. Diabetic animals treated with hydromethanolic extract as well as ethyl acetate and n-butanol fraction shown significant (*P*<0.001) effect on serum protein, cholesterol and triglyceride level (Table 2). In comparison to other extracts, n-butanol fraction was found to be more potent in normalizing the blood lipids and protein level. Proximate chemical analysis reveals the presence of steroids, flavonoids, tannins and carbohydrates in hydromethanolic extract where as ethyl acetate and n-butanol fractions were found to be very rich in flavonoid content. Thus, flavonoids from *Cassia auriculata* Linn. may be responsible for its antidiabetic potency. This study, therefore, indicates n-butanol as a potent fraction responsible for antihyperglycemic effect of *Cassia auriculata* flowers in alloxan-induced diabetes.

**TABLE 1:** EFFECT OF EXTRACT AND FRACTIONS OF *CASSIA AURICULATA FLOWERS* ON BODY WEIGHT AND BLOOD SUGAR LEVEL

Groups	Body weight (g)		Blood sugar level (mg/dl)		
		
	Before commencement of treatment	After end of the treatment	Normal	Alloxan treated	After extract/drug treatment
Normal control (Non-diabetic)	191.5±1.54	190±1.41	79.5±9.5	----	82.5±1.9
Diabetic control (Untreated)	188.8±0.92	175.6±1.92	83.2±5.6	291.2±1.6	----
Phenformin (0.04 g/kg)	183.6±1.67	182.6±1.25	90.5±8.6	372.0±2.1	141.8±1.5*
Hydromethanolic extract (0.20 g/kg)	216.5±5.02	210.6±4.1	86.5±0.9	293.2±1.6	201.8±2.6*
n-Butanol fraction (0.20 g/kg)	187.0±1.77	185.23±1.52	83.3±0.8	283.2±1.6	88.9±1.5*
Ethyl acetate fraction (0.20 g/kg)	206.0±1.31	201.2±1.29	88.6±0.9	289.6±1.9	127.2±1.5*

The observations are given as mean±SEM, n= 6

* p<0.01, compared with diabetic control group

**TABLE 2:** EFFECT OF EXTRACT AND FRACTIONS OF *CASSIA AURICULATA* FLOWERS ON BIOCHEMICAL PARAMETERS

Groups	Biochemical parameters			
	
	Cholesterol (mg/dl)	Triglyceride (mg/dl)	Total protein (g/dl)
Normal control (Non-diabetic)	67.2±1.1	95.8±1.7	7.1±.0.1
Diabetic control (Untreated)	179.3±1.7	186.2±1.3	4.4±.0.5
Phenformin (0.04 g/kg)	73.2±1.3*	112.9±1.0*	7.9±0.1*
Hydromethanolic extract (0.20 g/kg)	84.5±3.4*	125.7±1.3*	6.7±0.2*
n-Butanol fraction (0.20 g/kg)	74.6±1.2*	110.1±1.3*	7.4±0.1*
Ethyl acetate fraction (0.20 g/kg)	85.5±1.2*	121.6±2.5*	7.4±0.2*

The observations are given as mean±SEM, n= 6

* *P*<0.01 compared with diabetic control group
